# Mitochondrial DNA T7719G in *tRNA-Lys* gene affects litter size in Small-tailed Han sheep

**DOI:** 10.1186/s40104-017-0160-x

**Published:** 2017-04-01

**Authors:** Xiaoyong Chen, Dan Wang, Hai Xiang, Weitao Dun, Dave O. H. Brahi, Tao Yin, Xingbo Zhao

**Affiliations:** 1 College of Animal Science and Technology, China Agricultural University, Beijing, 100193 China; 2 Institute of Animal Science and Veterinary of Hebei Province, Baoding, 071000 China

**Keywords:** Association, Haplotype, Mitochondria, Non-synonymous mutation, Reproduction, Sheep

## Abstract

**Background:**

In farm animals, mitochondrial DNA (mtDNA) effect on economic performance remains hot-topic for breeding and genetic selection. Here, 53 maternal lineages of Small-tailed Han sheep were used to investigate the association of mitochondrial DNA variations and the lambing litter size.

**Results:**

Sequence sweeping of the mitochondrial coding regions discovered 31 non-synonymous mutations, and the association study revealed that T7719G in mtDNA *tRNA-Lys* gene was associated with litter size (*P* < 0.05), manifesting 0.29 lambs per litter between the G and T carriers. Furthermore, using the mixed linear model, we assayed the potential association of the ovine litter size and haplogroups and multiple-level mtDNA haplotypes, including general haplotypes, assembled haplotypes of electron transport chain contained sequences (H-ETC), mitochondrial respiratory complex contained sequences (H-MRC) and mitochondrial genes (H-*gene*, including polypeptide-coding genes, rRNA genes and tRNA genes). The strategy for assembled mitochondrial haplotypes was proposed for the first time in mtDNA association analyses on economic traits, although none of the significant relations could be concluded (*P* > 0.05). In addition, the nuclear major gene *BMPR1B* was significantly correlated with litter size in the flock (*P* < 0.05), however, did not interact with mtDNA T7719G mutation (*P* > 0.05).

**Conclusions:**

Our results highlight mutations of ovine mitochondrial coding genes, suggesting T7719G in *tRNA-Lys* gene be a potentially useful marker for selection of sheep litter size.

**Electronic supplementary material:**

The online version of this article (doi:10.1186/s40104-017-0160-x) contains supplementary material, which is available to authorized users.

## Background

Mitochondria are responsible for ATP production in the electron transport chain (ETC) in cells. The ETC consists of five mitochondrial respiratory complexes (MRCs), of which complex I, complex III, complex IV and complex V are constructed by both mitochondrial and nuclear encoded proteins, and complex II is entirely encoded by nuclear genes. Mitochondrial genome codes 13 polypeptides, 2 rRNAs and 22 tRNAs [1]. Since the first report of mitochondrial DNA (mtDNA) effect on milk production traits in dairy cattle [2], mtDNA effects on various economic traits have been widely studied in livestock, including pigs [3, 4], dairy cattle [5, 6], beef cattle [7, 8], sheep [9, 10] and chickens [11, 12]. Litter size is one of the vital economic traits for animal breeding and production, which has been studied for decades. For sheep, the nuclear gene, *BMPR1B* was identified as one of causative genes for sheep prolificacy [13], and has been widely used in sheep breeding. For the mtDNA effect on litter size, researchers reported the association with ewe litter size among haplogroups in an Afec-Assaf flock, but did not found the interaction with *BMPR1B* effect [10]. However, previous studies reported poor mtDNA effects [14–17], which made it necessary to uncover the genetic contribution of mtDNA for ewe litter size. In this study, Small-tailed Han sheep, a prolific breed of China, were used to investigate the association of litter size with mtDNA coding genes, in which non-synonymous mutations were considered as possible functional SNPs. Besides the non-synonymous mutation and haplogroup, assembled haplotypes of ETC-contained mtDNA sequences, MRC-contained mtDNA sequences, and mitochondrial genes were analyzed, respectively. The strategy for assembled mitochondrial haplotypes was proposed for the first time in association analyses. In addition, *BMPR1B* effect and interaction with mtDNA mutations were analyzed.

## Methods

### Animals

In total, 117 lambing Small-tailed Han sheep from 53 maternal lineages (families divided by female ancestors) of the same flock were performed blood sample collection, and recorded one or more times of litter size (Additional file 1: Table S1). All sheep were kept indoors year-round, and fed with mixed silage and hay to meet their nutritional requirements. Litter size (number of lambs born) was recorded, and full pedigree information was collected for all animals as reproductive management of the flock where ewes were mated with the selected rams after spontaneous estrus. All the ewes were genotyped for the *BMPR1B* at >2-month age.

### Genotyping of *BMPR1B* and sequencing of mitochondrial coding genes

Genomic DNA was extracted using the standard phenol/chloroform method [18]. The *BMPR1B* was genotyped using PCR-RFLP assay with the *Ava* II restriction enzyme [19]. Mitochondrial complete coding sequences were amplified by 17 primer pairs [20], and PCR products were sequenced in the Sanger method.

### Haplotype and haplogroup constitution

All non-synonymous mutations were used to constitute the haplotype and haplogroup. Considering the possible action for mitochondrial function, haplotypes were furthermore assembled by mtDNA sequence of ETC, MRCs and genes respectively. Haplotypes were determined by the online software FaBox [21], and haplogroups were constituted based on network analysis by Network 4.6.1.4 [22].

### Association analysis

Association analyses were carried out in the following mixed model by MIXED procedure in SAS software version 9.2 (SAS Institute Inc., Cary, North Carolina, USA).

$$ l s= y s+ parity+ ram+ BMPR 1 B+ mutations+ BMPR 1 B\times mutations+ ID+ E P+ e $$

In the model, the effects of lambing year-season (*ys*), parity number (*parity*), service ram (*ram*), *BMPR1B* genotype (*BMPR1B*), mtDNA mutations (*mutations*, including the effects of mtDNA non-synonymous mutations, haplotypes and haplogroups), the interaction between *BMPR1B* and mtDNA mutations (*BMPR1B × mutations*), the polygenic effect (*ID*), the permanent environmental effect (*EP*), and the random residual (*e*) were included. The response variable was the ewe litter size (*ls*). Each cell of these effects contained observations. The polygenic effect corrected the genetic background by the additive genetic relationship matrix, *i.e.* the pedigree information. The permanent environmental effect dealed with the repeated measurement data.

Inference about the interaction effect was made. If non-significant, that effect was dropped from the model and inference was made about the main effects. If significant, the cell means for the interaction became of interest.

When a set of statistical inferences were simultaneously considered, multiple comparisons were conducted by the false discovery rate (FDR) in the R project (R version 3.2.5) [23].

## Results

### Mutations in mitochondrial coding genes

In total, 95 mutations in mitochondrial coding genes were discovered (Additional file 1: Table S2), including 64 synonymous and 31 non-synonymous mutations (19 missense mutations in protein coding genes, 8 mutations in rRNAs, and 4 mutations in tRNAs respectively), which were illustrated in Table 1.

**Table 1:** Non-synonymous mutations in mitochondrial coding genes and corresponding effects on litter size

Gene	Nucleotide^a^	Codon mutation	Amid acid substitution	Significance^b^
*ND1*	T3543A	UCA → ACA	S → T	ns
*ND2*	T4208C	AUA → ACA	M → T	ns
*COII*	C7500A	CCC → CAC	P → H	ns
*ATP6*	A8039G	AAC → AGC	N → S	ns
	G8264C	GGA → GCA	G → A	ns
*COIII*	A9375G	AUA → GUA	M → V	ns
*ND4L*	C9974T	CCU → UCU	P → S	ns
	G10118A	GGU → AGU	G → S	ns
*ND4*	G10937A	GAC → AAC	D → N	ns
	G11045A	GUU → AUU	V → I	ns
*ND5*	G12571C	GGC → GCC	G → A	ns
	G13041A	GCA → ACA	A → T	ns
*ND6*	C13576T	CUC → UUC	L → F	ns
	T13588C	UAC → CAC	Y → H	ns
	C13777T	CAU → UAU	H → Y	ns
	C13789T	CAU → UAU	H → Y	ns
	T13837C	UCA → CCA	S → P	ns
	T13855C	UUC → CUC	F → L	ns
	A13876G	AUA → GUA	M → V	ns
*12SrRNA*	T281C	-	-	ns
	C291T	-	-	ns
	A538G	-	-	ns
*16SrRNA*	A1099T	-	-	ns
	T1112C	-	-	ns
	T2199A	-	-	ns
	C2443T	-	-	ns
	T2634C	-	-	ns
*tRNA-Tyr*	G5295A	-	-	ns
*tRNA-Lys*	T7719G	-	-	*
*tRNA-His*	C11606T	-	-	ns
*tRNA-Ser*	G11668A	-	-	ns

^a^Mutation positions were defined according to the ovine mitochondrial sequence (GenBank Accession nos.: AF010406)

^b^When a set of statistical inferences were simultaneously considered, multiple comparisons were conducted by the FDR using the R project. “ns” represents “not significant”, and “*” represents “significant” at the significant level of 0.05

### *BMPR1B* genotypes

For *BMPR1B* gene, 117 ewes were genotyped, including ++ genotype of 18 ewes, B+ genotype of 87 ewes and BB genotype of 12 ewes.

### Effects of mitochondrial haplotype and haplogroup on ovine litter size

**Fig. 1:**
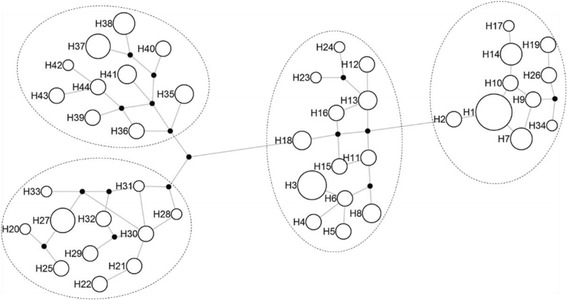
Neighbor-joining tree of different maternal pedigrees for haplogroup assignments by the 31 non-synonymous mutations

The 31 mitochondrial non-synonymous mutations assigned the Small-tailed Han sheep flock to 44 haplotypes (Additional file 1: Table S3), which were clustered into 4 haplogroups (Fig. 1). Using the mixed linear model, no significant effect on any haplotype or haplogroup was associated with litter size of Small-tailed Han sheep (*P* > 0.05), and the interaction between *BMPR1B* and haplotype or haplogroup also did not significantly affect litter size (*P* > 0.05), while the *BMPR1B* was positively associated with litter size (*P* < 0.05).

### Effects of missense mutations and haplotypes in mitochondrial protein coding genes on ovine litter size

Totally 35 H-ETCs were constituted by 19 missense mutations in protein coding sequences, while 15 missense mutations in MRCI assembled 33 H-MRCIs, and both 2 mutations in MRCIV and MRCV constituted 3 H-MRCIVs and 3 H-MRCVs respectively. Intensively, H-*gene*s were assembled by mutations of individual genes (Table 2 and Additional file 1: Table S4, S5 and S6). With mixed linear model analyses, no significant association was detected between litter size and the 19 non-synonymous mutations (*P* > 0.05) (Table 1), nor was any haplotype (*P* > 0.05) (Table 2). *BMPR1B* was strongly associated with litter size (*P* < 0.05) (Table 3), but the interaction of *BMPR1B* and mtDNA mutations was not remarkable (*P* > 0.05).

**Table 2:** Haplotypes constituted by mitochondrial non-synonymous mutations in multiple levels and significant effects on litter size

Functional haplotype^a^	Contained gene	Contained mutation number	Haplotype number	Significance^b^
H	*ND1*, *ND2*, *ND4L*, *ND4*, *ND5*, *ND6*, *COII*, *COIII*, *ATP6*, *12SrRNA*, *16SrRNA*, *tRNA-Tyr*, *tRNA-Lys*, *tRNA-His*, *tRNA-Ser*	31	44	ns
H-ETC	*ND1*, *ND2*, *ND4L*, *ND4*, *ND5*, *ND6*, *COII*, *COIII*, *ATP6*	19	35	ns
H-MRCI	*ND1*, *ND2*, *ND4L*, *ND4*, *ND5*, *ND6*	15	33	ns
H-MRCIV	*COII*, *COIII*	2	3	ns
H-MRCV	*ATP6*	2	3	ns
H-*ND4L*	*ND4L*	2	4	ns
H-*ND4*	*ND4*	2	4	ns
H-*ND5*	*ND5*	2	3	ns
H-*ND6*	*ND6*	7	10	ns
H-*ATP6*	*ATP6*	2	3	ns
H-*12SrRNA*	*12SrRNA*	3	4	ns
H-*16SrRNA*	*16SrRNA*	5	15	ns

^a^The general haplotype (H) was assembled by all non-synonymous mutations to reflect the integrated characteristics of mtDNA coding regions. Subsequently, the ETC based haplotype (H-ETC) represented the general feature of all ETC-contained mtDNA sequences. The MRC based haplotype (H-MRC) inferred the integrated signal of particular MRC-contained mtDNA sequences. Here, H-MRC included three types, i.e. MRC-I, MRC-IV and MRC-V. The last was the gene-level haplotype (H-gene), which indicated the information of a particular gene-contained mtDNA sequence

^b^When a set of statistical inferences were simultaneously considered, multiple comparisons were conducted by the FDR using the R project. “ns” represents “not significant”, and “*” represents “significant” at the significant level of 0.05

**Table 3:** Effects of *BMPR1B* on litter size

Genotype	Ewe number	Parity number	Litter size (Means)^1^
++	18	69	1.4638^a^
B+	87	285	1.7474^b^
BB	12	47	2.1915^c^

^1^“Means” represents the arithmetic average on litter size of sheep with the genotype. The FDR method was used to conduct multiple comparisons, and means with different lowercase letters are different at the significant level of 0.05

### Effects of mutations and haplotypes in mitochondrial rRNA genes on ovine litter size

The 3 mutations in *12SrRNA* sorted the sheep flock into 4 haplotypes (H-*12SrRNA*), and the 5 mutations in *16SrRNA* constituted 15 haplotypes (H-*16SrRNA*) (Table 2 and Additional file 1: Table S7). Association analyses revealed that neither mutation nor haplotype in rRNA genes affected litter size (*P* > 0.05) (Tables 1 and 2). The *BMPR1B* genotype was remarkably associated with litter size (*P* < 0.05) (Table 3), however, was inconspicuously correlated to rRNA mutations (*P* > 0.05).

### Effects of mutations in mitochondrial tRNA genes on ovine litter size

There were 4 mutations in tRNA genes, and only one variation was observed in each of them (Table 1), therefore no haplotype was constituted. Notably, T7719G in *tRNA-Lys* affected litter size by 0.29 lambs per litter between the G (1.79) and T (1.50) carriers (*P* < 0.05). Even though the *BMPR1B* was in prominent correlation with litter size (*P* < 0.05) (Table 3), there was no interaction with T7719G (*P* > 0.05) (Additional file 1: Table S8).

## Discussion

In human diseases, mutations in mitochondrial coding genes led to changes of oxidative phosphorylation enzyme complexes [24]. It is rational to image the possible effects of mtDNA on livestock traits. In this study, the Small-tailed Han sheep were used to explore the correlation of mtDNA and ewe litter size.

In order to investigate the overall mtDNA effects, a novel strategy of assembling mitochondrial haplotypes based on biology functions is proposed. Extensively, there are four levels of mitochondrial haplotypes for mitochondrial biological functions. The general haplotype is assembled by all non-synonymous mutations to reflect the integrated characteristics of mtDNA coding regions. Subsequently, the ETC based haplotype (H-ETC) represents the general feature of all ETC-contained mtDNA sequences. The MRC based haplotype infers the integrated signal of particular MRC-contained mtDNA sequences. H-MRC includes four types, i.e. MRC-I, MRC-III, MRC-IV and MRC-V. The last is the gene level, which indicates the information of a particular gene-contained mtDNA sequence.

In the mixed linear model, unnecessary factors interfere with the estimation of interested factors, as the degree of freedom is wasted. Therefore, based on the thoughts of multiple models [10] and variable selection in a mixed linear model [25], a two-step method of environmental variable selection was put forward in the association analyses, in which non-significant environmental factors were ignored to improve accuracy of genetic estimation. In this study, the environmental factors included lambing month, parity of dam, and service ram, and the genetic factors, which we were interested in, included *BMPR1B* genotype, mtDNA mutations and their interaction. We started a full model (Model I) with all the environmental and genetic factors to test if environmental factors were noises. Results revealed that the effects of parity of dam and service ram were not significant on litter size. Subsequently, these two factors were excluded in the aims to construct an optimized model (Model II), which was used to test the significance of genetic factors, especially the effects of mtDNA mutations on litter size.

**Fig. 2:**
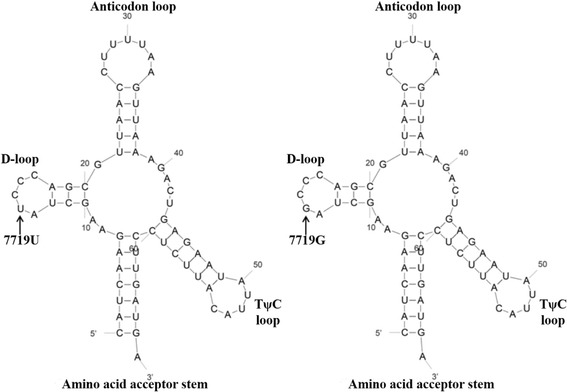
The predicted secondary structures of the *tRNA-Lys* for U7719G mutation

Many mitochondrial tRNA mutations in human were reported to be associated with wide range of pathological conditions [26], for example, deafness was correlated with mitochondrial *tRNA-Asp* A7551G mutation [27], hypertension was associated with mitochondrial *tRNA-Ile* A4263G mutation [28], and mitochondrial tRNA mutations might decrease in carcinoma hepatocyte [29]. In sheep, A7755G in *tRNA-Lys* was reported to affect litter size in an Afec-Assaf flock [10], while our study revealed that T7719G in *tRNA-Lys* was significantly associated with ewe litter size in Small-tailed Han sheep, with the means of 1.79 and 1.50 for the G and T carriers, manifesting a difference of 0.29 lambs per litter. Furthermore, the mutation was predicted to produce a transversion of U to G at DHU loop on 2D cloverleaf of the tRNA-Lys structure (Fig. 2).

## Conclusions

As a conclusion, we summarize the research procedures for mtDNA effects. Firstly, sweeping mitochondrial variations on maternal lineages; secondly, constituting the haplotype, haplogroup and assembled haplotypes; lastly, analyzing the association between mtDNA mutations (individual mutations, haplotypes and haplogroups) and interested traits. The present study discovered the mtDNA T7719G was linked with ewe litter size. For traits of low heritability, the marker assisted selection could increase the accuracy of breeding and selection. For the further study, the mtDNA T7719G should be put into post-association validation, and may become a genetic marker in the sheep breeding programs.

## Additional file

Additional file 1: Table S1. Detail information of used sheep. **Table S2.** Information of variations in mtDNA coding region of Small-tailed Han sheep. **Table S3.** The haplotype constituted by the 31 mitochondrial non-synonymous mutations in mitochondrial coding regions (H). **Table S4.** Assembled haplotypes of mitochondrial electron transport chain contained sequences(H-ETC). **Table S5.** Assembled haplotypes of mitochondrial respiratory complex contained sequences (H-MRCI, H-MRCIV, and H-MRCV). **Table S6.** Assembled haplotypes of protein coding genes (H-*ND4L*, H-*ND4*, H-*ND5*, H-*ND6*, H-*ATP6*). **Table S7.** Assembled haplotypes of rRNA coding genes (H-*12SrRNA*, H-*16SrRNA*). **Table S8.** Statistics for the number of observations in each cell of the interaction between *BMPR1B* and T7719G in* tRNA-Lys* and corresponding effects on litter size. (XLSX 33 kb)

## Abbreviations

mtDNA: Mitochondrial DNA
ETC: Electron transport chain
MRC: Mitochondrial respiratory complex
H: The haplotype constituted by all non-synonymous mutations in mitochondrial coding sequences
HG: The haplogroup classified by H
H-ETC: The assembled haplotypes of mtDNA electron transport chain contained sequences
H-MRC: The assembled haplotypes of mtDNA mitochondrial respiratory complex contained sequences, including H-MRCI, H-MRCIII, H-MRCIV, and H-MRCV, corresponding to complex I, III, IV and V, respectively
H-*gene*: The assembled haplotypes of mitochondrial genes, including polypeptide-coding genes, rRNA genes and tRNA genes
